# Production and Anti-Inflammatory Performance of PVA Hydrogels Loaded with Curcumin Encapsulated in Octenyl Succinic Anhydride Modified Schizophyllan as Wound Dressings

**DOI:** 10.3390/molecules28031321

**Published:** 2023-01-30

**Authors:** Lingyun Tu, Yifeng Fan, Yongfei Deng, Lu Hu, Huaiqing Sun, Bisheng Zheng, Dengjun Lu, Chaowan Guo, Lin Zhou

**Affiliations:** 1 School of Light Industry and Food Engineering, Guangxi University, Nanning 530004, China; 2 Guangdong Marubi Biotechnology Co., Ltd., Guangzhou 510700, China; 3 Guangdong Provincial Key Laboratory of Advanced Drug Delivery, Guangdong Provincial Engineering Center of Topical Precise Drug Delivery System, School of Life Sciences and Biopharmaceutics, Guangdong Pharmaceutical University, Guangzhou 510006, China; 4 School of Food Science and Engineering, South China University of Technology, Guangzhou 510641, China

**Keywords:** schizophyllan, octenyl succinic anhydride, curcumin, PVA, hydrogel, wound-healing

## Abstract

Amphiphilic polysaccharides can be used as wall materials and applied to encapsulate hydrophobic active chemicals; moreover, there is significant demand for novel medical high-molecular-weight materials with various functions. In order to prepare amphiphilic schizophyllan (SPG), octenyl succinic anhydride (OSA) was chosen to synthesize OSA-modified schizophyllan (OSSPG) using an esterified reaction. The modification of OSSPG was demonstrated through FT-IR and thermal analysis. Moreover, it was found that OSSPG has a better capacity for loading curcumin, and the loading amount was 20 μg/mg, which was 2.6 times higher than that of SPG. In addition, a hydrogel made up of PVA, borax, and C-OSSPG (OSSPG loaded with curcumin) was prepared by means of the one-pot method, based on the biological effects of curcumin and the immune-activating properties of SPG. The mechanical properties and biological activity of the hydrogel were investigated. The experimental results show that the dynamic cross-linking of PVA and borax provided the C-OSSPG/BP hydrogel dressing with exceptional self-healing properties, and it was discovered that the C-OSSPG content increased the hydrogel’s swelling and moisturizing properties. In fibroblast cell tests, the cells treated with hydrogel had survival rates of 80% or above. Furthermore, a hydrogel containing C-OSSPG could effectively promote cell migration. Due to the excellent anti-inflammatory properties of curcumin, the hydrogel also significantly reduces the generation of inflammatory factors, such as TNF-α and IL-6, and thus has a potential application as a wound dressing medicinal material.

## 1. Introduction

Skin, as the human body’s first line of defense [1], once damaged, can risk the exposure of subcutaneous tissues and organs to pathogens and water loss [2]. Every year, millions of people around the world die from infections due to a lack of effective wound care [3]. Wound dressings create a physical barrier between the external environment and the wound to prevent further infection, and some can even participate in the wound-healing process and promote wound repair [4]. However, wound healing is a long and complex biochemical process, which is divided into four main steps: hemostasis, inflammation, proliferation, and remodeling [5,6]. Therefore, the ideal wound dressing should provide a moist wound environment and have anti-inflammatory properties, whilst also promoting cell proliferation and migration. Many researchers are devoted to the development of materials with these characteristics. Thus far, many types of materials have been used in wound development and have been successfully commercialized [7,8,9]. Polysaccharides, such as chitosan [10] and dextran [11], have been widely used in wound dressing development because of their wide availability, affordability, minimal irritancy, and absorbability. In addition, polysaccharides can provide a wide range of structural parameters and properties for the manufacture of wound dressings, attributed to their multiplicity of molecular weight, charge, and chemical structure [12]. Among them, hydrogels based on polysaccharides are one of the most promising materials for wound dressing applications.

Schizophyllan (SPG) is an extracellular, naturally occurring, and high-molecular-weight polysaccharide produced by *Schizophyllum commune* with a 1,3-linked glucan backbone and a 1,6-linked D-glucose side chain at every third residue [13]. SPG has been applied as a biological-response modifier in clinical medicine [14], and some polysaccharides with similar structures to SPG have also exhibited good results, such as promoting cell proliferation [15], migration [16], and dermal contraction [17]. At the same time, SPG also exhibits certain anti-virus activities [18] and moisture properties, which can provide a continuous moist environment for wound healing because of its high water retention capacity. Additionally, the yield of SPG was significantly improved by bidirectional fermentation and the response surface methodology in our recent work [19]. The possible anti-aging activities of the fermentation supernatant have been illuminated [20]. Thus, the exploration of the multiple uses for SPG has become an interesting and challenging research focus. Nevertheless, it is not sufficient to construct an ideal wound dressing with SPG alone due to its limited anti-inflammatory activity. Curcumin has become very popular in the biomedical field, largely due to its antioxidant and anti-inflammatory effects [21]. The complex effect of polysaccharides and curcumin can improve the water solubility of curcumin and increase its bioavailability [22], and the hydrophobic modification of polysaccharides can further increase the loading of curcumin. Octenyl succinic anhydride (OSA), recognized as a green esterification agent, is a compound with high chemical activity due to its carbon–carbon double bond and carboxylic-acid-matching bond, with the advantages of being a less-toxic organic solvent, and can be easily applied in subsequent treatments [23,24]. The study of octenenyl succinic anhydride modified polysaccharide includes carrageenan [25], Arabic gum [26], and starch [27], which have all been well studied. In particular, octenenyl succinic anhydride-modified starch has been successfully applied in the food industry. However, to the best of the authors’ knowledge, there are no reports of octenenyl succinic anhydride-modified SPG (OSSPG).

In addition, SPG can form a gel in the presence of sorbitol or borax, but the gel is relatively weak and brittle [28]. A combination of two different polymer chains can lead to a double-network gel with high toughness and high stretchability. In detail, PVA is a hydrophilic, biocompatible, nontoxic, and biodegradable synthetic polymer [29]. Due to the hydrophilic functional groups in each molecular unit, PVA can form chemically and physically into a cross-linked hydrogel. Although introducing borax as a cross-linker into PVA hydrogel could significantly enhance the malleability, the mechanical properties and ductility were still poor. However, it has been shown that the addition of polysaccharides, such as nanocellulose [30] and xylan [31], to the system can improve the mechanical and tensile properties of the hydrogel. In this study, we combine the advantageous properties of SPG, curcumin, and PVA to generate a hydrogel for wound healing. The morphological, mechanical, anti-inflammatory, and wound-healing properties of the hydrogels were evaluated in vitro.

In this work, we synthesized an amphiphilic SPG by modifying it with octenyl succinic anhydride. The results of the infrared and thermogravimetric analyses showed that octenyl succinic acid was successfully grafted onto the SPG. The curcumin loading of the OSSPG was 2352 times that of curcumin in an aqueous solution, which provided a new strategy for the solubilization of hydrophobic substances. Meanwhile, a double network hydrogel with self-healing abilities and certain mechanical properties was prepared using the one-pot method. The self-healing, stretching ability, swelling, and water loss of the hydrogel were systematically investigated. In addition, the wound healing of the composite hydrogel dressing was evaluated through in vitro experiments. This work provides new insights into the use of OSSPG as wall materials for encapsulating complex and bioactive components, thus facilitating the development and application of OSSPG as a novel wound-healing material.

## 2. Results and Discussion

### 2.1. FT-IR Analysis

**Figure 1:**
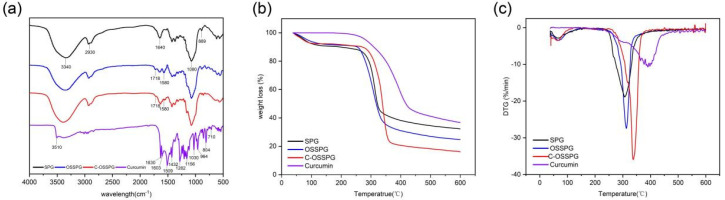
FT-IR spectra of SPG, OSSPG, C-OSSPG and Curcumin (**a**). Thermogravimetric analysis (**b**) and derivative thermogravimetry (**c**) of SPG, OSSPG, C-OSSPG, and curcumin.

The FTIR spectra were applied to determine the structural differences between SPG and OSSPG, and the results proved the successful esterification of SPG (Figure 1a). The wide peak at 3340 cm^−1^ in the spectrum of SPG originated from the O–H stretching of the hydrogen bonds [32]. The peaks at 2930 cm^−1^ and 1640 cm^−1^ are assigned to the C-H stretching and variable angle vibrations [33,34]. The peak at about 1080 cm^−1^ refers to the C–O linkage. Absorptions at 889 cm^−1^ are typical for β-D-glucose in a pyranose form [35]. As shown in Figure 1a, two new absorption bands have appeared in the spectrum of OSSPG in comparison to the spectrum of SPG. A sharp peak at 1718 cm^−1^ in the spectrum of OSSPG is attributed to the C=O stretching vibration of the carboxylic group of OSA [25]. The absorption band around 1580 cm^−1^ for OSSPG can be ascribed to the asymmetric stretch vibration of carboxylate (RCOO−) [36], which proves the successful esterification of SPG. The result was also in agreement with that of a previous study [37].

Curcumin is a polyphenolic substance present in an enol structure, with no obvious absorption peak at 1800–1650 cm^−1^. The hydroxyl group of curcumin had an absorption at 3510 cm^−1^ [38]. The C=O and C=C vibration at 1509 cm^−1^ and the stretching vibrations of the benzene ring showed sharp absorption bands at 1603 cm^−1^. The characteristic peak at 1283 cm^−1^ was due to the aromatic C–O stretching vibrations, and the peaks at 856 cm^−1^ were attributed to the C–O–C stretching vibrations of curcumin [39]. The characteristic peaks of C-OSSPG at 1718 cm^−1^ and 1580 cm^−1^ remained, and the phenolic hydroxyl characteristic peak at 3510 cm^−1^ and 1630 cm^−1^ disappeared due to the mask of the signal peak due to the embedding of curcumin into the hydrophobic core formed by the vector. The C-OSSPG did not change significantly compared to the OSSPG profile, indicating that no other chemical bonds were generated, and there were only intermolecular interactions between curcumin and OSSPG.

### 2.2. TG Analysis

As seen in Figure 1b, the TGA curves of SPG, OSSPG, C-OSSPG, and curcumin were investigated to reflect their thermal stability. SPG, OSSPG, and C-OSSPG had two observable independent stages of weight loss, respectively, indicating that the presence of different components leads to different degradation temperatures. Specifically, the dehydration stage of all of the samples, except curcumin, was mainly completed within 100 °C, which is attributed to polymer dehydration due to the fact that polysaccharides are highly hygroscopic due to their hydrophilic character. The weight loss related to water in SPG, OSSPG, and C-OSSPG at the first step (50–100 °C) was 9.39, 8.82, and 5.29%, respectively. The hydrophilic groups of the polysaccharide were replaced by the hydrophobic groups, resulting in a lower water absorption capacity. However, the free curcumin did not lose weight in the first stage, which is mainly due to the highly hydrophobic polyphenol of curcumin, containing almost no free water. In the second stage, drastic weight losses of 58.32% for SPG, 66.41% for OSSPG, 76.10% for C-OSSPG, and 63.23% for curcumin were observed. There is an obvious difference in the maximum decomposition temperature in the second stage. At illustrated in Figure 1c, the maximum decomposition temperatures reached 312.9 °C, 306.8 °C, 337.6 °C, and 390.7 °C for SPG, OSSPG, C-OSSPG, and curcumin, respectively. It was observed that the grafting of the octenyl succinate chains onto the SPG may have caused the lower degradation temperatures, which is illustrated by the fact that the introduction of the OS group negatively affected the thermal stability of SPG. The decrease in the thermal stability was caused by the weakened hydrogen bonds between the hydrophobic alkenyl group and SPG, resulting in a lower required energy input for bond breakage, which is similar to Cheng’s report [40]. The decrease in the thermal stability was caused by the weakened hydrogen bonds between the hydrophobic alkenyl group and SPG, resulting in a lower required energy input for bond breakage [41]. However, the stability is increased when the OSSPG is complexed with curcumin, which demonstrates that there is an interaction between curcumin and OSSPG, and new complexes may form. Similarly, Li et al. [23] reported that curcumin lost its original crystal structure when it was integrated with the amphiphilic material, transforming into an amorphous state contained in the core of the micelle.

### 2.3. Loading Capacity (LC) of Curcumin

**Figure 2:**
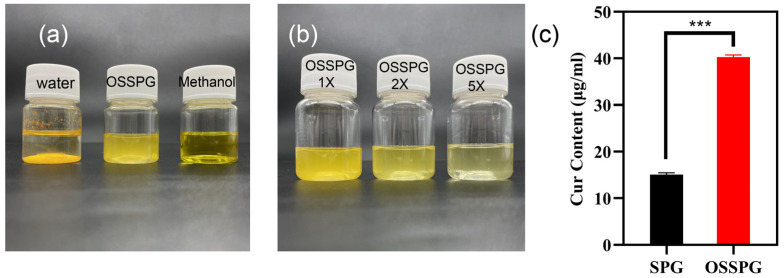
The visual appearance of curcumin in water, OSSPG solution and Methanol (**a**). The visual appearance of C-OSSPG solution of different concentrations, C-OSSPG 2X and 5X, represent two- and five-fold diluted C-OSSPG solutions, respectively (**b**). Loading capacity of curcumin in SPG and OSSPG (**c**). In the chart, *** represents *p* < 0.001.

Curcumin is a water-insoluble polyphenol substance with a solubility of only 17 ng/mL in water. As shown in Figure 2a, curcumin precipitates completely in water but appears bright yellow in methanol solution and translucent dark yellow in OSSPG solution. After two- and five-times dilution of C-OSSPG, the OSSPG solution loaded with curcumin gradually became transparent and clear (Figure 2b), indicating that the overall solution was relatively uniform, and no precipitation occurred after two weeks of placement. Figure 2c shows that the embedding amount of curcumin in SPG was only 15 μg/mL, while the embedding amount of curcumin in OSSPG solution was up to 40 μg/mL. This may be due to the hydrophobic nature of OSSPG, which can form hydrophobic interactions and hydrogen bonding with curcumin [42]. It also appeared between the curcumin and hydrophobicity modified starch, and the solubility of the curcumin increased by approximately 1670-fold after encapsulation [43]. This may be due to the ability of amphiphilic polysaccharides to self-assemble into micelles for hydrophobic ingredient encapsulation [23]. Therefore, this study further confirms that the polysaccharide modified with OSA facilitates the loading of hydrophobic substances.

### 2.4. Properties of Hydrogel

**Figure 3:**
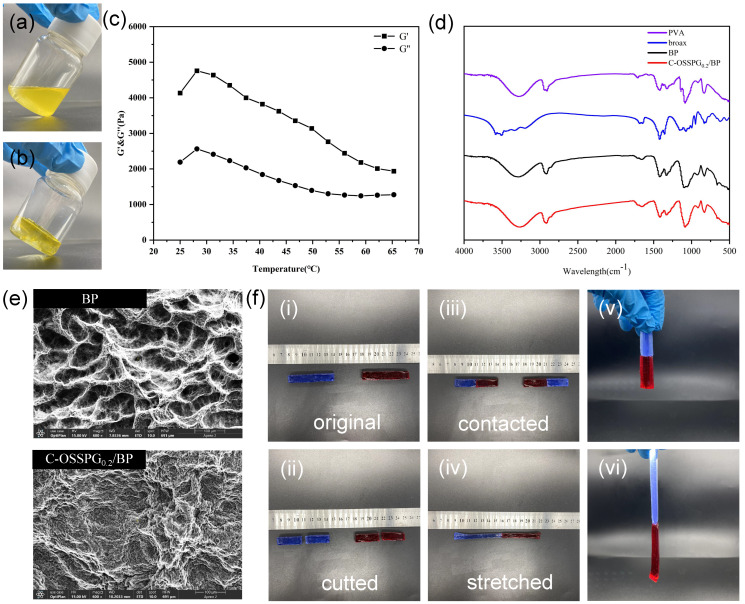
Before hydrogel formation (**a**). After hydrogel formation (**b**). Temperature (T) sweep experiment at t = 25–65 °C of C-OSSPG_0.2_/BP hydrogel (**c**). FT-IR spectra of PVA, borax, BP and C-OSSPG_0.2_/BP hydrogel (**d**). The SEM image of BP and C-OSSPG_0.2_/BP (**e**). The relevant physical properties of the C-OSSPG_0.2_/BP hydrogel (**f**).

Figure 3a,b shows the visual images of C-OSSPG_0.2_/BP before and after hydrogel formation, respectively. Figure 3c shows that the G′ value of the hydrogel is greater than the G″ value, indicating that the C-OSSPG_0.2_/BP hydrogel exhibited elastic-like behavior [44]. Additionally, the hydrogel can maintain the gel state between 25 and 65 °C.

The FT-IR spectra for PVA, borax, BP, and the C-OSSPG_0.2_/BP hydrogel are presented in Figure 3d. It is worth noting that the absorption peaks for BP and C-OSSPG_0.2_/BP hydrogel arising at 832 and 665 cm^−1^ were assigned to the B-O stretching from the residual B(OH)_4_^−^ and the bending of the B-O-B linkages within the borate network [31], respectively. The asymmetric stretching of B-O-C from the hydrogel showed discernible absorption peaks at 1330 and 1420 cm^−1^ after the addition of B molecules within the PVA hydrogels [45]. All of these results confirmed the presence of cross-linking between borate ions and OSSPG or PVA chains.

The study of the surface morphology was performed using SEM to detect the cross-linking pattern of the BP and C-OSSPG_0.2_/BP hydrogels. The porous structure of the BP hydrogel is shown in Figure 3e. However, the surface morphology of the C-OSSPG_0.2_/BP hydrogel changed. As a result of the intermolecular interaction between OSSPG and PVA, the majority of the pore space in the BP hydrogel was filled with OSSPG, resulting in smaller and denser pores [46].

Figure 3f shows the relevant physical properties of the C-OSSPG_0.2_/BP hydrogel. Two colored 6 cm columnar-shaped hydrogels were cut into two pieces and then recombined at the fracture surfaces. Two pieces of fractured hydrogel healed autonomously and rapidly after 1 min of self-healing, without any external intervention, at room temperature. This is due to the presence of dynamic boronic ester linkages in the hydrogel that can satisfy the rapid healing of the hydrogel [47]. Moreover, to verify the mechanical properties of the reconstituted hydrogel, the hydrogel was fully stretched; the healed hydrogel can be stretched up to 13 cm, which is two times longer than the original. As presented in Figure 3f, no breaks in the healed hydrogel were observed when lifted from one end, indicating excellent stretchability and superior self-healing abilities.

### 2.5. Swelling Abilities

**Figure 4:**
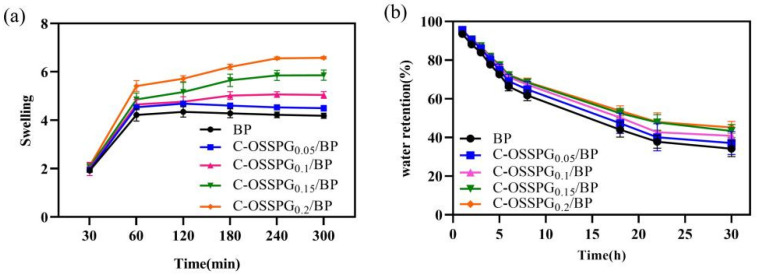
Swelling rate of the five kinds of hydrogels (**a**). Water retention of the five kinds of hydrogels (**b**).

The influence of different hydrogels on the swelling performance was analyzed. Figure 4a shows that the different C-OSSPG proportions of the composite hydrogels have similar swelling processes. Due to PVA’s inherent ability to absorb water, all of the hydrogels reached a swelling balance after 180 min in deionized water, and the swelling rate reached at least five times. This indicates that C-OSSPG hydrogels can quickly absorb extra body fluid from wounds. The swelling properties of the hydrogels cross-linked by borax in our work is better than the hydrogels of physical cross-linking in a previous study [48]. The swelling ratio was increased with the C-OSSPG content at the fixed PVA and borax content. Additionally, the water absorption of the C-OSSPG_0.2_/BP hydrogel can reach six times its weight, more than 1.3 times that of the BP hydrogel. The reason for this might be that OSSPG has abundant hydrophilic groups [49], such as hydroxyl and carboxyl, which can be easily hydrated with water molecules. The remarkable swelling ability of the C-OSSPG_0.2_/BP hydrogel ranks this material as a promising wound dressing.

### 2.6. Water Retention

Maintaining a moist environment is also critical for wound healing. Lightly moist environments can promote wound healing [50], and hydrogel dressings with higher water retention rates could reduce the replacement frequency, enabling quicker healing, less pain, and increased cost savings. According to the water retention data in Figure 4b, the addition of C-OSSPG favors the water retention of the hydrogel, showing similar results to a previous study [51]. The SEM image of the hydrogels indicated that the addition of OSSPG can lead to smaller and more compact holes, thus reducing the water evaporation rate. Moreover, all of the hydrogels still retained 35% to 50% water after 30 h, and the hydrogel containing 0.2% OSSPG could retain 45% water. This shows that the C-OSSPG_0.2_/BP hydrogel has good water retention. It is ascertained that PVA can retain a certain amount of water, and the addition of C-OSSPG increased the number of hydrophilic hydroxyl groups, which can be more closely combined with water for hydrogels.

### 2.7. Cytotoxicity of Hydrogels

**Figure 5:**
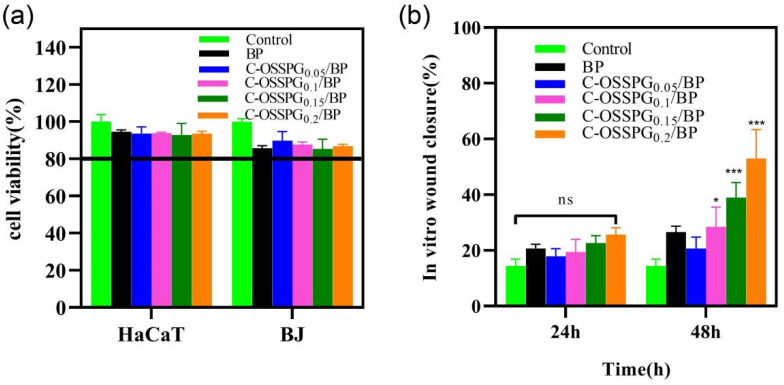
Cell toxicity of five hydrogels against HaCat and BJ cells (**a**). The effects of the BJ cell migration after treatment by five hydrogels for 0 h, 24 h, and 48 h (**b**). In the chart, ns represents *p* > 0.05, * represents *p* < 0.05, and *** represents *p* < 0.001.

Cytotoxic studies are needed to avoid toxicity in normal cells. Wound healing is a complex physiological process that requires the synergy of multiple cells during healing [52]. Keratinocytes and fibroblast cells all play important roles in wound-repair processes [53,54]. In the cell culture media, the hydrogel eventually degrades, releasing elements such as boron. The instantaneous concentration of the boron release was smaller than 100 ppm, which would not cause cell toxicity, according to the work of H. Wang [55]. In our study, the toxicity of the different hydrogels to two types of cells was mainly determined (Figure 5a). Although all of the hydrogels showed statistically significant differences in cellular activity compared to the negative control, the viability of the cell still reached 80% of the negative control. According to the International Standard ISO 10993-5 (2009), reducing cell viability by more than 30% is considered a cytotoxic effect. The results show that the C-OSSPG/BP hydrogel has biocompatibility and is a good candidate to be used as a wound dressing. We have tried to increase the content of polysaccharides, but the cytotoxicity results showed that nearly 30% of the cells died. The detailed reason is not clear, but may be associated with the high viscosity of high-molecular-weight polysaccharides.

### 2.8. In Vitro Wound-Healing Assay

**Figure 6:**
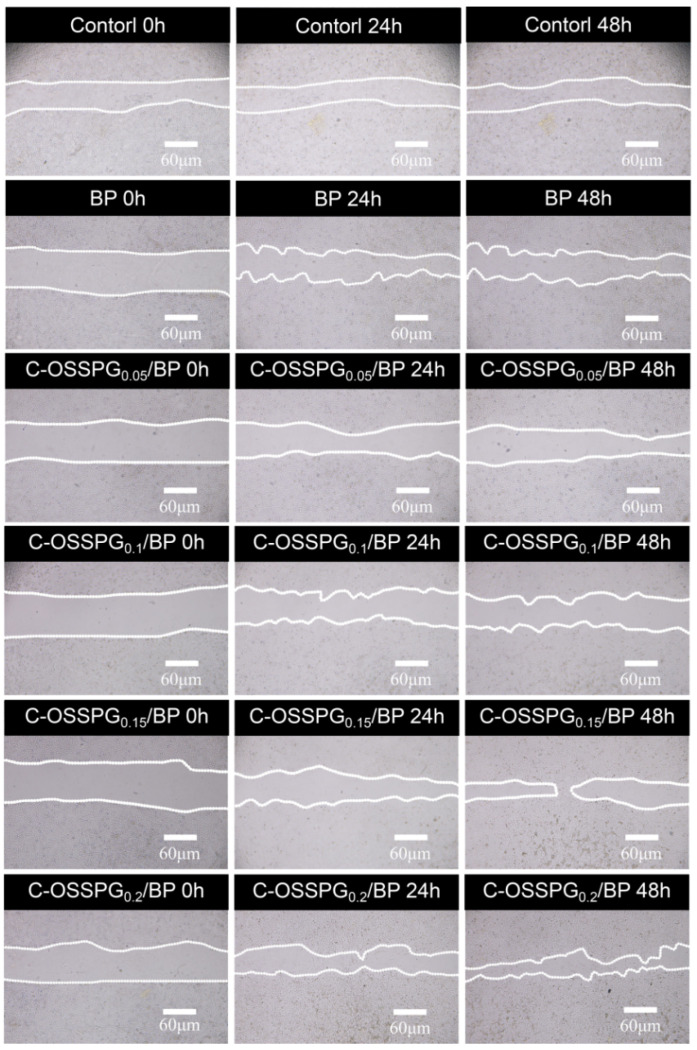
The picture of BJ cell migration after treatment by five hydrogels for 0 h, 24 h and 48 h.

Fibroblasts not only play a pulling role in the wound healing process, but part of them can also differentiate into myofibroblasts, which can secrete ECM and better promote wound healing [56]. Figure 5b shows the influence of the hydrogel on wound healing, which was evaluated using an in vitro scratch wound assay with fibroblasts. After 24 h, the upper and lower parts of the cells were close to each other (Figure 6). The fibroblasts advanced toward the opening to close the scratch wound by roughly 27.14% after a 24 h exposure to C-OSSPG_0.2_/BP, but promoted the healing of the wound without any significant differences compared to the control group. After 48 h, the C-OSSPG_0.2_/BP and C-OSSPG_0.15_/BP hydrogels promoted the in vitro wound closure rate significantly (*p* < 0.01). This shows that the hydrogels containing C-OSSPG at concentrations of 0.2 wt% and 0.15 wt% had excellent cell-healing-promoting effects on the BJ cells, thus accelerating wound healing. However, wound healing is a complex process, so further investigation may be required before its application to clinical use. Considering the appropriate water storage capacity, excellent water absorption, and cell migration ability, the C-OSSPG_0.2_/BP hydrogel was chosen to further study their anti-inflammatory properties.

### 2.9. Anti-Inflammatory

**Figure 7:**
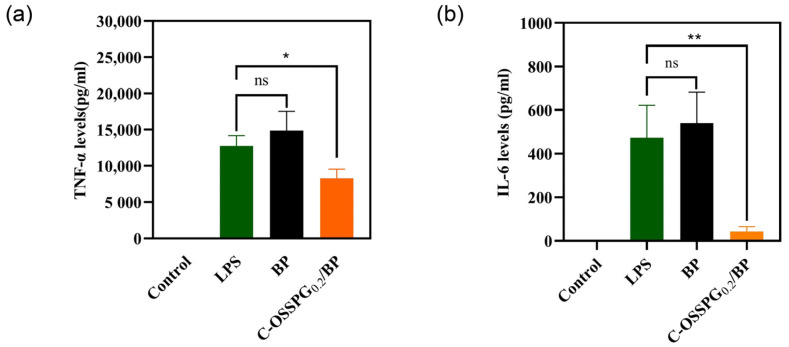
Effects of C-OSSPG_0.2_/BP hydrogels on inflammatory cytokine production, including TNF-α (**a**) and IL-6 (**b**). In the chart, ns represents *p* > 0.05,* represents *p* < 0.05, ** represents *p* < 0.01.

Inflammation is an early innate immune response to tissue damage and is important in the wound-healing process [57]. Increased anti-inflammatory activity is the key to accelerating wound healing. However, excessive inflammatory factor secretion can be detrimental and lead to chronic wounds that are difficult to heal and, in some cases, even cancer. As a typical representative of a polyphenolic compound, curcumin has strong anti-inflammatory [58], antimicrobial [59], and antioxidant bioactive activities and has been used in China for centuries to treat inflammatory and chronic diseases. A plethora of studies has shown that curcumin is also a good wound-healing drug that can promote cell healing [60,61]. As shown in Figure 7a, RAW 264.7 cells treated with only LPS were considered as a group for inflammatory models. Compared with a group of inflammatory models, the TNF-α contents released from the RAW 264.7 cells reduced by 34.76% after being treated with the C-OSSPG_0.2_/BP hydrogel, although they increased by 16.5% after being treated with the BP hydrogel. This suggests that the use of the hydrogel without C-OSSPG alone causes further inflammation. Similar to TNF-α, the C-OSSPG_0.2_/BP hydrogel can effectively inhibit the production of IL-6 (*p* < 0.01); the IL-6 contents released from the RAW 264.7 cells reduced by 81.7% after being treated with the C-OSSPG_0.2_/BP hydrogel (Figure 7b). The results showed that the C-OSSPG_0.2_/BP hydrogel could effectively inhibit the production of inflammatory factors, such as TNF-α and IL-6, thus accelerating the anti-inflammatory repair phase of the wound and facilitating wound healing.

## 3. Conclusions

In conclusion, OSSPG was successfully prepared to improve the hydrophobicity of SPG for use in curcumin-encapsulated applications, and the final experiment showed that the curcumin loading capacity of OSSPG increased to 40 μg/mL. The FT-IR and TG analysis showed that OSSPG forms a novel complex with curcumin. Therefore, it could be an ideal candidate for hydrophobic drug delivery in the biomedical field. Subsequently, we designed a hydrogel composed of C-OSSPG, PVA, and borax, which has good tensile properties and self-healing abilities. The swelling, water retention, and in vitro cell experiments of the hydrogel were also investigated. By adding C-OSSPG, the swelling of the hydrogel and water retention abilities were improved. The results show that the hydrogel containing 0.2 wt% C-OSSPG could significantly increase the cell migration rate of BJ cells and suppress the production of TNF-α and IL-6. Therefore, the multifunctional properties of the C-OSSPG_0.2_/BP hydrogel make it an excellent candidate for bioactive wound dressings.

## 4. Materials and Methods

### 4.1. Materials

Curcumin (≥95.0% purity) was purchased from Sigma-Aldrich Corp. (St Louis, MO, USA) and 2-Octen-1-ylsuccinic anhydride (99.0% purity) was purchased from Shanghai yuanye Bio-Technology Co., Ltd. (Shanghai, China). Schizophyllan was obtained from the fermentation products of *Schizophyllum commune* from alcohol precipitation. PVA (Mowiol^®^ 117, Mw = 145,000 g/mol) was supplied by Aladdin Biochemical Technology Co., Ltd. (Shanghai, China). High-glucose DMEM medium and trypsin were provided by U.S. Gibico Corporation. Fetal bovine serum was provided by Hangzhou Sijiqing Bioengineering Materials Co., Ltd. (Hangzhou, China). All the other chemical materials were of minimum analytical grade. The enzyme-linked immunosorbent assay (ELISA) kit for tumor necrosis factor-alpha (TNF-α) and interleukin-6 (IL-6) was supplied by Neobioscience Technology Company (Shenzhen, China).

### 4.2. Octenyl Succinic Schizophyllan Synthesis

The SPG was prepared by the method described by Deng et al. [20]. The final fermentation products, including SPG, were centrifuged at 8000 rpm for 10 min to remove most of the strains. Then, ethanol (96%, *w*/*v*) was gradually added to the obtained liquid supernatant for the precipitation of crude polysaccharides. After standing for 12 h at 4 °C, the precipitated SPG was collected through centrifugation and finally freeze-dried.

**Figure 8:**
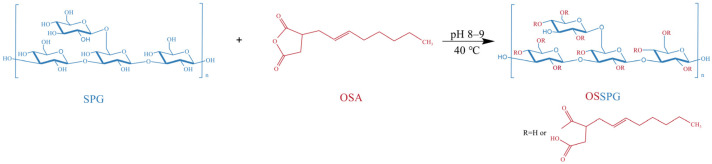
Schematic presentation of OSSPG.

The OSSPG was synthesized according to a previously reported method for starch octenyl succinate, with slight modifications [62]. Precisely weighed freeze-dried SPG powder was dissolved in distilled water. After full hydration, the solution was stirred at 80 °C for 2 h and centrifuged at 3000 rpm for 10 min to remove the precipitate. The content of schizophyllan was adjusted to 2.5 mg/mL by adding distilled water. After that, the pH of the solution was adjusted to 8.0–9.0 using 2 M NaOH, which was applied to control the pH of the system between 8.0 and 9.0 during the whole reaction process. Ethanol-diluted octenyl succinic anhydride was slowly added to the aqueous SPG solution and stirred continuously at 40 °C for 2 h. The OSSPG was obtained from the esterification reaction between the hydroxyl groups of SPG and OSA under alkaline conditions (Figure 8). The pH of the aqueous solution was adjusted to 6.5 by the addition of 1 M HCl at the end of the reaction. To eliminate the unreacted OSA, the final product was washed more than three times with 96% ethanol. The OSSPG was obtained by redissolving the precipitate in water and dialyzing (MWCO, 12 kDa) against deionized water for 24 h and finally freeze-drying.

### 4.3. Substitution Degree of OSSPG

The substitution degree (DS) of OSSPG was measured using the HPLC method, as described by Qiu et al. [63]. The standard curve of the trans-2-octenyl succinic acid obtained through high-performance liquid chromatography was y = 2638.4x − 21.067 R^2^ = 0.9991, where y is the OSA content (mg/mL), and x is the peak area of trans-2-OSA in the liquid chromatogram. The substitution of the final modified polysaccharide was 0.023.

### 4.4. Preparation of the OSSPG/Cur Inclusion Complex

Then, 10 mg of curcumin was added to a 50 mL centrifuge tube containing 10 mL of OSSPG at a concentration of 0.2%. Then, the solution was homogenized at 10,000 rpm for 5 min using a homogenizer (IKA T18 digital ULTRA TURRAX, Frankfurt, Germany) to make full contact between the curcumin and the modified polysaccharide. Finally, the solution was centrifuged at 10,000 rpm for 10 min to remove the free curcumin, and the supernatant was collected for further analysis. The filtrate containing the polymeric solution was freeze-dried and stored in the dark at 4 °C for characterization. The final sample was named C-OSPPG, where C signifies the presence of curcumin.

### 4.5. Loading Capacity

The loading capacity (LC) of the curcumin was detected based on a previously published method [64]. A double volume of methanol was added to 1 mL of the SPG/OSSPG solution loaded with curcumin and then spun strongly under a vortex for 5 min and centrifuged at 5000 rpm for 5 min. Lastly, the absorbance value was measured by taking the supernatant at 422 nm using a UV spectrophotometer, and the total curcumin content was calculated according to the methanol-curcumin standard curve. A calibration curve was used to calculate the content of curcumin (y = 0.1344x − 0.0246, R^2^ = 0.9990).

### 4.6. Synthesis of Hydrogels

**Figure 9:**
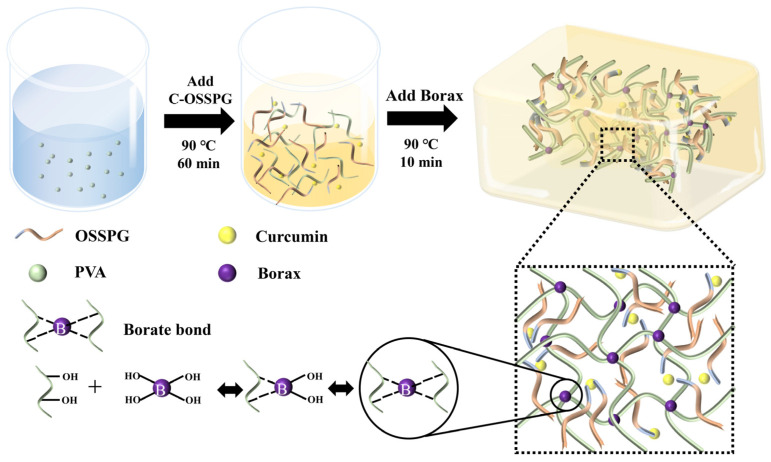
Schematic illustration of the preparation process of C-OSSPG/BP hydrogel.

The hydrogels were synthesized using a one-step method, described by Siqi Huang [44]. The hydrogels with 10% PVA, 1.5% borax, and the desired amount of C-OSSPG were prepared through a one-pot reaction. First, PVA powder was dissolved in deionized water (90 °C) by stirring for 1 h to form a transparent and uniform solution (20%). Afterward, different concentrations of the same volume of C-OSSPG solution and borax were added to the PVA solution. Finally, all of the mixed solutions were frozen at −25 °C for 8 h, and the hydrogel was obtained after thawing at room temperature for at least 10 h in the mold. Figure 9 describes the preparation process of the C-OSSPG/BP hydrogel. For comparison, a hydrogel excluding C-OSSPG was prepared and marked as PB, and the PB hydrogels containing C-OSSPG with concentrations of 0.05%, 0.10%, 0.15%, and 0.20% were labeled as C-OSSPG_0.05_/PB, C-OSSPG_0.1_/PB, C-OSSPG_0.15_/PB, and C-OSSPG_0.2_/PB, respectively.

### 4.7. Characterization Analysis

FT-IR analysis: The chemical structures of the SPG, OSSPG, and C-OSSPG were investigated using an FT-IR spectrometer (Nicolet IS50, Waltham, MA, USA). The spectra were recorded in the wavelength range of 500–4000 cm^−1^ with a resolution of 4.0 cm^−1^. The samples were dried at 105 °C for 12 h before analysis to avoid the interference of moisture.

TG analysis: The thermal stability of the SPG, OSSPG, C-OSSPG, and curcumin were analyzed using a TGA-851e (Mettler Toledo, Schwerzenbach, Switzerland). The samples with a mass between 3 and 5 mg were placed in an alumina crucible at a detection temperature of 50–600 °C, a nitrogen flow rate of 20 mL/min, and a heating rate of 10 °C/min, using nitrogen as a protective gas. An empty pan, sealed in the same manner, was used as a reference.

SEM analysis: The morphological investigations of BP and the C-OSSPG_0.2_/BP hydrogels were performed using a scanning electron microscope after vacuum-coating the samples with plat. A small piece of dried hydrogel was fixed onto the SEM stubs and sputter-coated with a thin layer of Au prior to examination.

Rheological measurements: The rheological behaviors of the C-OSSPG/BP hydrogels were analyzed with a modular rheometer. The sample was made into a cylindrical shape, with a diameter of 15 mm and a thickness of 6 mm. The temperature (t) was increased at T = 25–65 °C, and the scanning strain (γ) = 0.5%, 0.5 rad/s, to determine the change in the storage modulus and the loss modulus of the sample due to the temperature.

### 4.8. Macroscopic Self-Healing Test of C-OSSPG_0.2_/BP Hydrogel

The C-OSSPG_0.2_/BP hydrogel with a diameter of 10 mm and a length of 60 mm was prepared in advance. To intuitively show the self-healing effect, two C-OSSPG_0.2_/BP hydrogels were stained with methyl blue and Congo red, respectively. The hydrogel was cut into two pieces, and then the different colors of the two pieces were re-spliced together and stored at 25 °C for 1 min to allow them to self-heal into a complete hydrogel. The tensile capacity of the hydrogel was also tested by a simple stretching of the re-healed hydrogel. Photographs of the hydrogels under different situations were taken.

### 4.9. Swelling

The swelling properties of the freeze-dried hydrogel samples were evaluated. Then, all of the samples were immersed in PBS buffer (pH~7) at 37 °C until the gel reached the equilibrium state of swelling. The hydrogel was removed, and the excess solvent on the surface was removed by blotting quickly with absorbent paper, and then weighed. The swelling ability of the hydrogel can be formulated using a swelling ratio; the swelling ratio was calculated as follows:

Swelling Ratio (SR) = (W_s_ − W_d_)/W_d_,

where W_d_ is the weight of the dried hydrogel and W_s_ is the weight of the swollen hydrogel.

### 4.10. Water Evaporation Rate

The prepared hydrogel was immersed in distilled water. After it reached swelling equilibrium, the hydrogel was placed in an incubator at 50 °C for 24 h under a humid atmosphere of 50%. The weight of the hydrogel was measured at regular intervals until completely dry. The water evaporation rate of the hydrogel can be formulated using the water retention equation as follows:

Water retention = (W_s_ − W_m_)/(W_s_ − W_f_) × 100,

where W_s_, W_m_, and W_f_ are the starting weight, measured weight, and last weight of the hydrogel, respectively.

### 4.11. Cell Cytotoxicity Assay

The cytotoxicity of the hydrogels was evaluated using the ISO 10993-5 method [65]. The in vitro cytotoxicity of the hydrogel was evaluated against the human Fibroblast cell line (BJ) and HaCat cell line (HaCat) using the CCK8 assay [66]. The cells were cultured in Gibco’s medium, supplemented with 10% fetal bovine serum and 1% penicillin/streptomycin. The cell suspensions were seeded at a concentration of 1.0 × 10^5^ cells/mL in 96-well plates, at 100 μL per well. Before the experiments, various synthesized hydrogels were sterilized using UV radiation for 120 min. Subsequently, hydrogel-extracted liquid solutions were prepared by soaking the hydrogels (1 g) in DMEM (4 mL) containing serum at 37 °C for 24 h. The culture media were aspirated, then all of the cells were incubated with extracting liquid solutions for the different hydrogels for 24 h. Finally, the treatment solution was aspirated, and 100 μL of 10% CCK8 solution was added to every well and then incubated in the cell incubator for half an hour before measuring the absorbance value at 450 nm. Cytotoxicity was calculated as the percentage of absorbance in wells with solution-treated cells to that of the control cells (100%), as follows:

Cell Viability(%) = (OD_hgydrogel_/OD_control_) × 100

### 4.12. In Vitro Wound-Healing Assay

The ability of BJ to migrate to the wound environment from adjacent areas determines the speed of wound healing. The wound-healing potential of the hydrogels was assessed using an in vitro wound-healing assay [67]. For this purpose, the BJ cells were seeded at a density of 1.5 × 10^5^/well onto a coated 24-well plate in 10% FBS-DMEM at 37 °C and 5% CO_2_ to obtain a monolayer of cells. Then, a sterile gun tip was used to draw a line through the monolayer of cells, and then they were washed with PBS. The cells were incubated with the treatment solutions for the different hydrogels or 2% FBS-DMEM (untreated control) for 48 h. The migration rates were observed and photographed under microscopy. The percentage of the closed area was analyzed by using ImageJ, and then compared with the control.

### 4.13. Anti-Inflammatory

RAW 264.7 cells were seeded in 24-well plates at a density of 4 × 10^5^ cells/well for 24 h. After removing the cell culture medium, the cells were then stimulated with LPS at 1 µg/mL, followed by treatment with the treatment solutions for the different hydrogels. After incubation for 24 h, the culture medium was collected for the determination of TNF-α and IL-6 levels. The levels of TNF-α and IL-6 were determined using a mouse enzyme-linked immunosorbent assay (ELISA) kit according to the manufacturer’s instructions. The TNF-α and IL-6 results for each sample were calculated from their respective standard curves.

### 4.14. Statistical Analysis

All of the tests were performed in triplicate, unless otherwise indicated, and the values are expressed as the mean ± standard deviation. One-way analysis of variance was performed to evaluate the significance at *p* < 0.05 by Duncan’s multiple range tests. In the chart, * represents *p* < 0.05, ** represents *p* < 0.01, and *** represents *p* < 0.001.

## Data Availability

Not application.

## Abbreviations

SPG	Schizophyllan
OSA	Ocenyl succinate anhydride
OSSPG	Octenylsuccinic schizophyllan
C-OSSPG	Curcumin loaded octenylsuccinic schizophyllan
DS	Substitution degree
HPLC	High Performance Liquid Chromatography
FT-IR	Fourier Transform Infrared spectroscopy
TG	ThermoGravimetry
TGA	Thermogravimetric Analysis
SEM	Scanning Electron Microscope
LC	Loading capacity
BP	Hydorgel containing Borax and PVA
C-OSSPG_x_/BP	Hydorgel containing Curcumin loaded octenylsuccinic schizophyllan, Borax, and PVA, x represent the concentration of curcumin loaded octenylsuccinic schizophyllan
BJ	Fibroblast cell line
HaCaT	HaCat cell line
DMEM	Dulbecco’s modified eagle medium
FBS	Fetal Bovine Serum
RAW 264.7	leukemia cells in mouse macrophage
LPS	Lipopolysaccharide
TNF-α	tumor necrosis factor-α
IL-6	Interleukin-6
